# Adapting Behavioral Interventions for Social Media Delivery

**DOI:** 10.2196/jmir.5086

**Published:** 2016-01-29

**Authors:** Sherry Pagoto, Molly E Waring, Christine N May, Eric Y Ding, Werner H Kunz, Rashelle Hayes, Jessica L Oleski

**Affiliations:** ^1^ Department of Medicine Division of Preventive and Behavioral Medicine University of Massachusetts Medical School Worcester, MA United States; ^2^ Departments of Quantitative Health Sciences and Obstetrics & Gynecology University of Massachusetts Medical School Worcester, MA United States; ^3^ Digital Media Lab University of Massachusetts Boston Boston, MA United States

**Keywords:** social media, behavioral interventions, health behavior, online social networks

## Abstract

Patients are increasingly using online social networks (ie, social media) to connect with other patients and health care professionals—a trend called peer-to-peer health care. Because online social networks provide a means for health care professionals to communicate with patients, and for patients to communicate with each other, an opportunity exists to use social media as a modality to deliver behavioral interventions. Social media-delivered behavioral interventions have the potential to reduce the expense of behavioral interventions by eliminating visits, as well as increase our access to patients by becoming embedded in their social media feeds. Trials of online social network-delivered behavioral interventions have shown promise, but much is unknown about intervention development and methodology. In this paper, we discuss the process by which investigators can translate behavioral interventions for social media delivery. We present a model that describes the steps and decision points in this process, including the necessary training and reporting requirements. We also discuss issues pertinent to social media-delivered interventions, including cost, scalability, and privacy. Finally, we identify areas of research that are needed to optimize this emerging behavioral intervention modality.

## Introduction

The term Web 2.0, coined in 1999, refers to Web technology that allows users to interact and create content in virtual communities, which represents a divergence from static websites that only allow users a passive role as consumers of information. Social media is a broad example of Web 2.0 and refers to online social networking sites such as Facebook, Twitter, Reddit, Pinterest, and Instagram, as well as blogs and message boards, all of which are tools that allow users to engage with one another and generate their own content. Social media usage has exploded in recent years such that it is nearly ubiquitous, with 89% of US adults now using the Internet and the majority of those (74%) having at least one social network account [1,2]. Prevalence of social media use is highest among younger adults. The Pew Internet Project reported in January 2014 that 89% of 18-29-year-olds use online social networking sites compared to 82% of 30-49-year-olds, 65% of 50-64-year-olds, and 49% of adults aged 65 years or older [2]. Similar rates of use of social media have been reported for men (74%) and women (76%), and among blacks (75%), Hispanics (80%), and whites (70%) [3]. The vast majority of users log into their preferred networks daily [4]. In fact, Facebook recently reported that US adult users spend, on average, 40 minutes a day on Facebook [5].

Online social network use is no longer limited to keeping in touch with friends and family; many users now seek and exchange information about health [6], parenting [7], and a wide variety of other topics. The 2011 Pew Internet Survey found that 34% of Internet users have read a commentary or experience about health or medical issues on a website or blog [8]. People are not just in search of health information on the Internet, but are also in search of other patients [9]. The US Department of Health and Human Services Chief Technology Officer, Susannah Fox, labeled this emerging trend as “peer-to-peer health care” and explains, “Patients know things—about themselves, about treatments—and they want to share what they know to help other people.” Fox refers to “peer-to-peer health care” as “the most exciting innovation in health care today” [10]. That social media provides a means for health care professionals to communicate with patients and for patients to communicate with each other presents an opportunity to use this modality to deliver behavior change programs.

**Figure 1:**
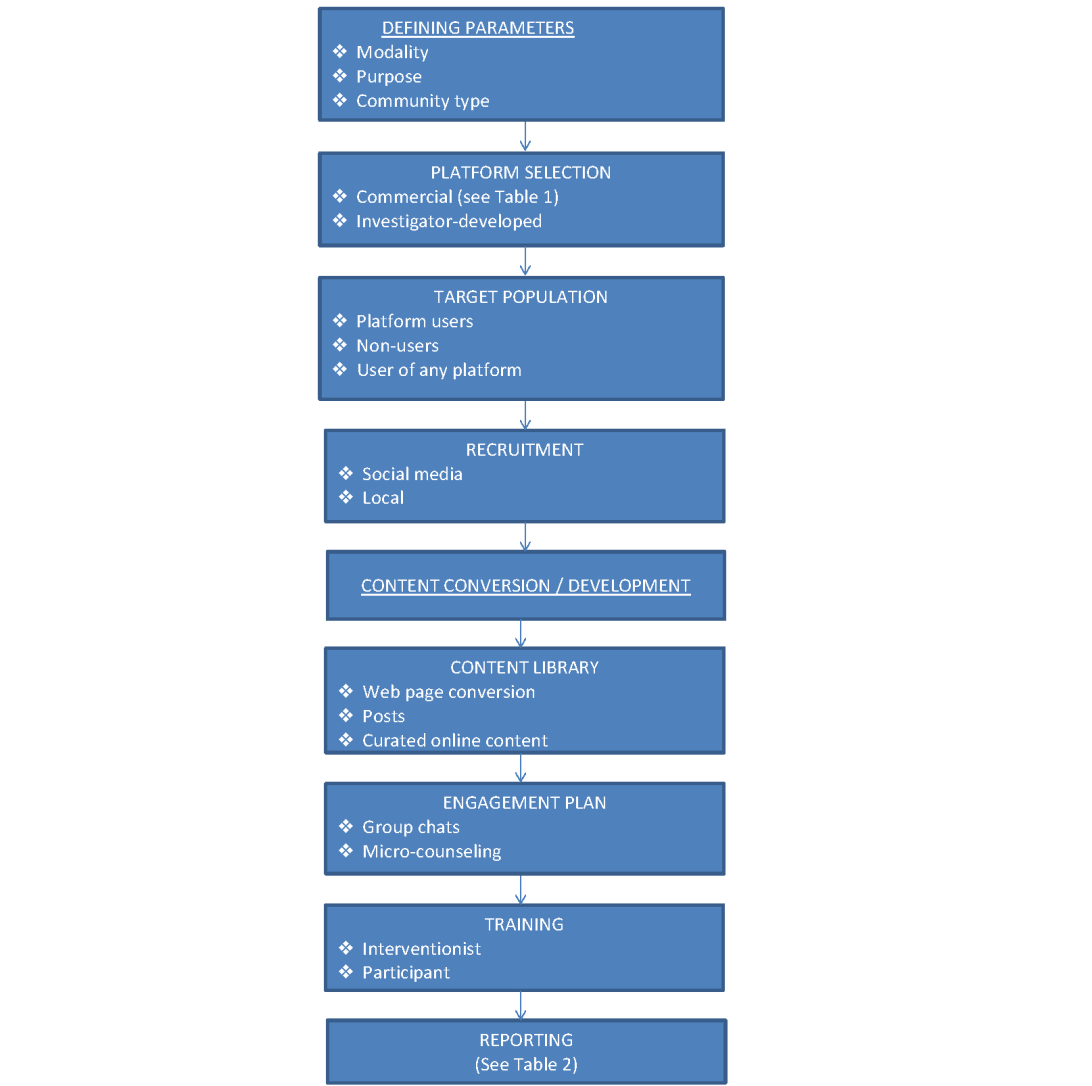
The process of adapting a behavioral intervention for social media delivery.

Scheduling constraints, family obligations, and lack of transportation negatively impact attendance in traditional in-person behavior change interventions [11]. The burden of the traditional intervention modality centers around the clinic visits (eg, high frequency and long duration) [12]. Delivering behavioral interventions via an online social network circumvents many of these barriers by reducing or eliminating visits. Using online social networks as a behavioral intervention modality allows us to take advantage of how embedded online social networking is in people’s lives. People can participate as part of their usual social media routine rather than carve out large chunks of time from their usual activities to participate. The absence of scheduled meetings allows people to engage flexibly each day, when the time is best for them. Advice, support, and cues can be provided in the moment, when participants need it the most. The ability to be “in the participant’s pocket” and deliver real-time counseling and support has great potential for changing behavior.

Using online social networks to deliver behavioral interventions is a relatively new idea, thus much work with regard to design, content, implementation, and reporting of trial outcomes and engagement is needed. In this paper, we discuss the process by which investigators can translate behavioral interventions for social media delivery. We present a model (see Figure 1) that describes the steps and decision points in this process, including the necessary training and reporting requirements. We also discuss issues pertinent to social media-delivered interventions, including cost, scalability, and privacy. Finally, we identify areas of research that are needed to optimize this new behavioral intervention modality.

## Defining the Parameters of the Social Media-Delivered Intervention

### Online Social Network as Primary Delivery Modality Versus Supportive Adjunct

In the context of behavioral interventions, an online social network may be (1) the primary modality of intervention, (2) one modality in a multimodal intervention, or (3) a supportive adjunct to an intervention that is primarily utilizing another modality (eg, visits, telephone, mobile app, and text messaging). When an online social network is the primary intervention modality, all intervention content is delivered via the online social network. This means all intervention content must be in a format that is consistent with formats typically used in that online social network. For example, on Twitter, posts are limited to 140 characters and often include links to webpages with more information. On Facebook, no character limit is in place and use of links, videos, and pictures is common. On Instagram, all content is in the form of photos or videos, and on YouTube, all content is video.

When an online social network is one modality in a multimodal intervention, some, but not necessarily all, key active ingredients of the behavioral intervention are delivered via the online social network. In this type of intervention, the content meant for social media delivery would need to be converted into a format that is consistent with content typically circulated in the target online social network.

When the online social network is an adjunct to an intervention that is delivered primarily via another modality, it might provide resources and information to users but not active ingredients of the intervention. Still, in this scenario, all resources (eg, links and tips) and information need to be converted into an appropriate format for the target online social network. For successful translation, the research team must be familiar with not only the features and capabilities of the online social network, but also with the norms of their target audience on that social network. Clearly reporting the role of the online social network in the intervention and the origin and purpose of the content disseminated via the network is essential.

### Purpose of the Online Social Network

The purpose of the online social network may be to provide a means to deliver behavioral counseling; to provide supplemental information about behavioral strategies originally delivered via a different intervention modality (eg, clinic visits); to maximize attendance and retention in the intervention (eg, post reminders for meetings and follow-ups); to provide resources (eg, a list of local gyms for a physical activity study); to provide a place for participants to communicate, connect, and support each other; or any combination of the above. A major decision in intervention planning is whether the purpose involves facilitating participant engagement, information dissemination, or both [13].

When engagement is a purpose of the online social network, an engagement plan is needed. When information dissemination is the sole purpose of the online social network, efforts to elicit engagement are less important. In this case, a static website or email distribution list might also serve this purpose. The critical difference between a website and an online social network is the ability to facilitate user engagement. An advantage of an online social network over a static website is the ability to insert an intervention into participants' pre-existing social media feeds. This will only be the case, though, if the participants are regular users of the target social media platform. The combination of engagement and information dissemination may be the most effective approach, given studies showing that engagement in an online social network is associated with better outcomes on clinical end points [14-16].

### Host- Versus User-Generated Content

In designing a social media-delivered intervention, matching the type of intervention to the purpose (eg, behavioral counseling platform, public health campaign, or information only) is a necessary consideration. Content in social media-delivered interventions may be host generated [17], such that the host generates all content and pushes it to users; user generated [18], such that users generate all content; or host and user generated [19,20], such that content is created by both the host and users. Host-generated feeds are unidirectional, such as in the case of public health campaigns, which involve a feed of information relating to a specific topic. Examples include the US Centers for Disease Control and Prevention (CDC) Twitter feeds (eg, @CDC_cancer) and the Skin Cancer Foundation Facebook page [21]. Users can comment on host posts and reply to each other’s comments. Communities characterized primarily by user-generated content are typically started by a host who builds a forum for users to interact with one another, but does not provide content and does not typically interact with users (eg, PatientsLikeMe.com). Alternatively, a community may be initiated by a user for similar users to exchange information (eg, Fitbloggin Facebook page by a weight-loss blogger for weight-loss bloggers). Communities that include content that is both host and user generated are bidirectional such that both hosts and users generate content and interact with one another. Bidirectional communication is important when the purpose of the intervention is to be able to generate conversations, answer participant questions, help them solve problems, and/or provide behavioral counseling. Participants should be clear on the type of community to which they have been invited (ie, host and/or user generated) and the expectations for engagement.

## Social Network Platform Selection

### Overview

Investigators can choose a commercial social networking platform, a commercial app that has a social networking function, or they may create their own social network platform. In terms of commercial platforms, many are freely available and have large user bases, for example, Facebook and Twitter. These two platforms have been in existence for 11 and 9 years, respectively, and consequently have had the benefit of long periods of refinement, user buy-in, and proven usability. Selecting the commercial platform that is most used by the target population will result in more openness to use and more experienced users, which may reduce engagement barriers. Investigators are referred to the Pew Survey of Internet and American Life report on the demographics of users of the most popular social media sites [22]. Alternatively, investigators may elect to utilize the social network capabilities of commercial mHealth apps. Many mobile apps (eg, Endomondo and MyFitnessPal) have social networking features that can be used to engage with participants individually and see data they have recorded using the mobile app. Finally, investigator-designed social network platforms, as in Brindal et al [23], can also be used when commercial platforms do not have the functionality to meet the intervention specifications.

Several considerations go into choosing the best platform. An investigator-designed social network requires technical expertise and overhead costs for development and maintenance. Developing an engaging user interface requires a high level of technical expertise, cost, and development time. In a recent systematic review of weight-loss studies using online social networks, the authors explained that discussion boards and chat rooms used in investigator-designed websites lack the “sophisticated, user-friendly, vibrant platforms that incorporate a rich, pleasing, graphical environment” that are characteristic of mainstream online social network platforms [24]. Such an approach might be best matched for interventions that cannot be feasibly implemented using commercially developed platforms. An advantage of commercial platforms is that they are free and the technical skills required for use are becoming ubiquitous among many populations. A commercial platform that is heavily used by the target population (eg, targeted according to age, sex, and ethnicity) may not require extensive participant training. Software for managing, collecting, and analyzing social media data on many commercial platforms is also widely available and often free. However, researchers need to be aware of the limitations and challenges of using existing commercial online social networks, including privacy concerns, changing use policies, and changing data access policies. For example, Facebook at any time can change their application programming interface (API), which is the protocol that describes how different software programs can interact with Facebook. This may affect how data is extracted from Facebook and software compatibility. Data that was once accessible via certain means can become instantly inaccessible via those means. Consideration of the advantages and disadvantages of different platform options is an important step early in intervention development. The best platform for a particular behavioral target, intervention content, and target population may be inappropriate for other purposes. Table 1 describes the top nine commercial social media platforms [25-37].

**Table 1:** Characteristics of existing online social media platforms.

Platform	Year founded	Number of users	Medium of posts	Character limit	Private messages (yes/no)	Privacy functions allow creation of private groups (yes/no)	Chat function (yes/no)
Facebook	2004	1.44 billion [25]	Text, video, images	63,206 [26]	Yes	Yes	Yes
Twitter	2006	302 million [27]	Text, video, images	140	Yes	Yes	No
Pinterest	2010	72.8 million [28]	Text, video, images	500 [29]	Yes	Yes (private pin boards)	No
Snapchat	2011	100 million [30]	Video, images	31 for captions	Yes	No	Yes
LinkedIn	2002	364 million [31]	Text, images	600 [32]	Yes	Yes	Yes
Instagram	2010	300 million [33]	Video (<15 sec), images with captions	2200 for captions	Yes	No, but can send content to specific recipients	No
Google+	2011	300 million [34]	Text, video, images	100,000 [35]	Yes	Yes	Yes
Tumblr	2007	420 million users, 246 million blogs [36]	Text, video, images	No limit in blog posts; 250 in replies; 500 in “ask messages”	Yes	Yes (private group blog)	No
Vine	2013	40 million [37]	Video	N/A^a^ (clips limited to <6 sec)	Yes	No	No

^a^N/A: not applicable.

### Facebook

Facebook has two formats—fan pages and groups—that can be used for behavioral intervention delivery. Fan pages are public, where feeds can be followed when a user hits the “like” button of the fan page. These are ideal for public health campaign approaches. Groups, on the other hand, can be *public*, *private* (ie, only invited members can see content), or *secret* (ie, only invited members can see content, and existence of page is hidden) and can be used to generate conversations among a smaller group of people. A *secret* group is ideal when the investigators wish to keep all aspects of the intervention completely private.

An advantage of Facebook for behavioral intervention delivery is that it is the most popular social networking site, with 74% of US adults currently having an account. Participants may already be logging in regularly, which will bring them in regular contact with the intervention. Facebook also has settings in which communities can be created that allow users to participate privately even if their main account is public. Users can participate without their online friends being aware they are a member of the group. This might result in fewer privacy concerns and greater intervention receipt and participation. A drawback of Facebook is that it is not currently possible to change the organization of content. All exchanges appear on the “wall,” or main feed of the group page, including exchanges between two users. This can result in a busier program feed or deter one-on-one interactions when participants do not feel comfortable having a side conversation appearing on the group wall. Another drawback is that the feed in a private group is organized in order of posts that have received the most recent interactions, as opposed to the chronological order of posts. This setting is not modifiable and, as a result, important content may fall to the bottom of the feed on days with heavy posting, making it difficult for participants to locate intervention content. Facebook allows the moderator to “pin” a single post to the top of the wall to circumvent this issue. Another disadvantage is that laypersons may not trust or understand Facebook’s privacy settings, which can affect their interest in participating in the study [38].

A challenge specific to using fan pages on Facebook is that of intervention receipt. Facebook adjusts the number of posts from a fan page occurring in a user’s feed to the level of interaction on that page by the user using a proprietary formula. This means that a participant with low initial engagement on the page will receive fewer updates from the page in their newsfeed and this may continue to decline as their engagement declines. Investigators can still boost receipt and engagement by analyzing audience engagement frequently and tailoring the social media strategy according to what the audience engages with most, as discussed elsewhere [39]. This does not occur in a *secret* group. Facebook settings are subject to change, thus investigators are encouraged to review current settings at the beginning of the study, test the setting before engaging study participants, and keep track of any setting changes during the study. We refer investigators to Facebook Help Center, which provides a wealth of information about settings and privacy.

An advantage of Facebook is that it is a way to leverage participants' close social ties given that Facebook is used to connect to existing friend networks. This may be useful when doing family-based interventions or interventions targeting groups of people who are connected socially. While secret groups on Facebook do not leverage social support from participants’ Facebook friends, engaging close social ties may not always be helpful. Our previous work showed that for weight loss, social media users felt their Facebook friends were less of a source of weight-loss social support and more of a source of negativity relative to their weight-loss community on Twitter [40]. Thus, the ability to separate a health-focused online community from users’ personal communities may be an advantage when it comes to certain health behaviors. An alternative approach is to allow participants to invite their trusted Facebook friends into the intervention. As long as such individuals have consented, this approach could take advantage of social support from both close and loose social ties [41].

### Twitter

Twitter, an online social network that allows users to communicate via 140-character-or-less posts, is used by 23% of Americans [2]. Twitter has been used to deliver behavioral interventions in several studies [42,43]. The vast majority of Twitter users (88%) communicate publicly [44], meaning their tweets are viewable by anyone on the Internet. However, Twitter has a *protected account* setting that allows users to communicate privately. When an account is *protected*, the only users who can view the content are those who have been approved by the user. Private groups can be created on Twitter by having users set their accounts to *protected,* and by only following each other. This feature makes it highly conducive to facilitating confidential discussions in behavioral intervention groups. Avatars and pseudonyms can also be used to anonymize participants' bios to fully protect participants’ privacy. Unlike Facebook, creating a private group on Twitter requires the user to use *protected* privacy settings on their accounts, and in most cases, the user may prefer not to use their main account to participate in the intervention. The latter approach may be ideal because the intervention feed will then not get lost in the highly populated newsfeeds of experienced users. The Twitter app makes switching between accounts possible with a single click, as opposed to requiring logging out of one account and then into the other, as is the case with Facebook.

Users’ newsfeeds on Twitter are organized chronologically, as opposed to by most recently engaged posts as in Facebook. Twitter allows users to send private direct messages (DMs) to other users, including group facilitators. This allows users to connect on a one-on-one basis and is an advantage over Facebook interventions, where private messages can only be passed between users who are “friends”; however, being a member of a secret Facebook group does not require a user to be “friends” with the other members. Group facilitators should be cautious, though, that too much private messaging with participants could end up moving instructive conversations out of the group, which can increase the time needed to manage the group.

### Other Social Media Platforms

Other popular social media platforms include Instagram, Pinterest, YouTube, Vine, and Snapchat. Instagram involves sharing of images and videos, while YouTube and Vine involve sharing of videos. Pinterest involves sharing of links that are organized on “boards” that are decorated by images extracted from links. Clicking on the image takes the user to the link. Snapchat is a platform in which users can take photos and videos, add text and drawings, and send to selected users who can then view it for a time limit between 1 and 10 seconds. Multiple platforms can also be used in the same intervention. For example, videos in a Vine or YouTube feed can be shared on Facebook and/or Twitter. An alternative to commercial platforms is an investigator-designed platform that meets the specific needs of the intervention. In this case, content can be pushed to participants’ main social media feeds via share buttons to take advantage of participants’ social media presence on commercial platforms.

## The Target Population

In designing an online social network-delivered intervention, it is important to consider the social media experience of the target population. If the online social network is providing active intervention ingredients, ensuring that all participants have access to the online social network is necessary to maximize intervention receipt. Different subgroups, as defined by age, sex, cultural group, or other characteristics, may contain more frequent users of certain commercial media platforms. Recruiting individuals who are not active users or who have no experience engaging in a social media platform may result in their not receiving the full dose of the intervention. Some studies recruit both current users of the target platform and also individuals who are willing to open an account to participate in the study [45,46]. Even though participants might agree to sign up for an account on the target platform to participate in the study, the opportunity to embed the intervention into a pre-existing habit is lost with this target population. Instead, the intervention requires the development of a social media habit, which may or may not occur. Their log-ins may be solely for research participation and so this may mean fewer opportunities for them to be exposed to intervention content relative to regular users who will see it during their usual social media activities, even when their interest in the intervention is waning.

Other studies take a more conservative approach and limit study entry criteria to current users of the target social media platform [17,18,47-53]. Given that 61% of Facebook users surveyed reported they take breaks from using Facebook up to several weeks at a time [54], some studies have even more stringent entry criteria regarding use patterns [20,55]. For example, in one study, participants were required to be daily users of Facebook [20], and in another, participants were required to use Facebook for at least 30 minutes per day [55]. When using novel platforms, restricting participants to those with social media experience may reduce usability issues. Our previous work revealed that participants who were recruited as nonusers engaged minimally, even when staff provided instruction on how to use the online social network platform [43]. To ensure that users are familiar with the social networking site, they may be recruited directly from the site. For example, Facebook ads can be used for study recruitment for a fee. On Twitter, recruitment ads can be disseminated via tweets for free. Advertisements can target specific subgroups by using keywords or hashtags frequently used by that community. “Influencers”—people that represent a specific community and have a large following among that community—can also be engaged and/or incentivized to disseminate study advertisements [56].

## Content Conversion

Delivery of intervention content via an online social network requires transforming communication into a format that is consistent with how people communicate in online social networks. For example, in traditional intervention delivery modalities, content is delivered via printed handouts and/or a manual that provides the foundation for counselor-led discussions. However, content in online social networks is typically in the form of brief posts that sometimes include graphics, videos, or links to articles. One study found that information about contraceptives shared over Facebook—in video, graphic, and game format—led to higher knowledge scores than when information was distributed via pamphlet [57]. This shows that information shared via social media has the potential to be even more effective than print formats.

Posts with a graphic, video, or link to an article typically include a brief headline describing the content to attract viewers to read and/or click on it. Individuals and/or concepts in graphics, videos, and other media should reflect the target population. For example, an intervention targeting Latino moms should utilize images and videos that feature Latino moms in the preferred language of the population. Most online discussions are not scheduled, but rather happen spontaneously in the form of comments/replies or hitting a “like” or “favorite” button to indicate agreement. Online discussions are asynchronous, meaning a question may be posted by a user at one time and then answers by other users may appear throughout the day and sometimes into the next day, but not typically longer. Prior to the intervention, behavioral content should be converted into a content library that aligns with how users interact on the target social media platform. To this end, we recommend that a study team member is a regular and active user who can educate the team about the norms of the target platform.

## Content Library

### Overview

A content library is organized in a similar fashion as a treatment manual, but the content itself is in a different format. Content can include articles written by investigators that are posted on a website, links to other online resources (eg, recipes), brief posts that introduce links or videos (eg, “Check out these 5 ways to squeeze exercise into your day! Which will you try this week?”), infographics, images, gifs (ie, images with animation), videos, status updates, conversation starters, polls, event invitations, and chat topics.

### Documents-to-Webpage Conversion

Word processing documents (eg, .doc, .rtf, and .pdf) are rarely shared in online social networks and few networks even allow this capability. Instead, content can be broken down into a series of brief posts or into online articles. Online articles can be shared via links, a commonly shared format of information on social media. Using blogging software (eg, WordPress), lessons in a treatment manual can be converted into online articles with photos and videos embedded. Online articles are typically brief (ie, 800 words) and include images. Essentially, the treatment manual can be converted into a study blog/website that may or may not allow comments and can be publicly available or completely private (ie, accessible only via links, not search engines). Images used can be developed by the team, purchased from stock image websites, or copied from free stock image websites. Investigators should be aware that using images found via search engines may violate copyright laws, which has consequences especially if the treatment manual is published or sold.

### Creation of Posts

Once the treatment manual is converted into an online format, the next step is to create posts that introduce links in each post in a way that draws the users’ attention to the link. The goal is to achieve a high engagement rate, which includes all activities that a user can do with a post (eg, click on a link, “like” or “favorite” it, share it, or comment on it). The content in the link can only be effective if clicked on and read. Some social media platforms have character limits for posts (eg, Twitter) and others will limit how many characters can be viewed without necessitating an extra click. Although Facebook does not have character limits, a study of 11,000 Facebook pages found the optimal length of a post was about 120 characters, with longer posts getting lower click-through rates [58].

Intervention posts should also accurately convey what is to be found in the link, being careful not to bait users with sensationalism (eg, “Emotional eating no more! How to get over it for GOOD!”). The term “click bait” is used colloquially on social networking sites to refer to posts that exaggerate or sensationalize content in the link for the purpose of “baiting” people to click. Images can also be used to accompany posts as a way to graphically illustrate a concept or generate emotion. One social media marketing study found that 87% of posts with Facebook engagement had a photo [59]. A study by the social media marketing company, HubSpot, found that posts with photos get 53% more likes, 104% more comments, and 84% higher click-through rates than text-only posts [60]. A study of a smoking cessation campaign found that the most common type of engagement was comments on photos, but while participants found many posts motivating, some triggered the desire to smoke, which suggests that certain images might cue unhealthy behavior [61]. Finally, a study of the National Cancer Institute Facebook page found that posts with images received the most engagement relative to videos, links, and status updates [62].

Infographics are increasingly being used as an alternative way to depict research findings or other information via social media. Free software can be used to make infographics or companies can be hired to design professional-quality infographics. Infographics are available online as well. For example, the US Centers for Disease Control and Prevention has a gallery of infographics available to include in websites and online publications [63]. Videos may be another way to deliver content, and are commonly shared on social media platforms [64]. The majority of Internet users (78%) report watching videos online and 25% have uploaded videos [65]. Some platforms allow videos to be embedded into a post while in other platforms videos can be posted via links to their original source. However, investigators should avoid posting lengthy videos, as social media research reveals that the average length of time a user will watch a video is 2.7 minutes [66,67].

### Curating Evidence-Based Resources

Other resources that can be linked to in a social networking feed include links to reputable online resources. For example, in a weight-loss intervention, links to healthy recipes can be curated and distributed to participants. In a smoking cessation intervention, links to information and resources posted on the American Cancer Society webpage might be leveraged. In general, nonprofit scientific organizations typically have a great deal of curated content on their websites and social media feeds, which could be rich sources of evidence-based information and tools to support an intervention. Leveraging existing evidence-based content is an excellent use of available resources and a way to connect and acquaint users with legitimate sources of health information on the Internet, given the tremendous amount of false information available online. Investigators are encouraged to confirm that the link is active before posting, given occasional changes to URLs or removal of content on external websites.

## Engagement Plan

### Overview

The engagement plan should describe group size, frequency of posting, whether posts are automated, and a guide for how and how often interventionists should engage with participants. Group size is a consideration given that it is likely to influence engagement. Very small groups may have low engagement due to size, but then very large groups might have so much engagement that intervention content gets buried in the newsfeed. In 19 studies we found using Facebook to deliver behavioral interventions, group size ranged from 3 to 7282 participants [17-19,45,46,48-50,52,53,55,61,68-74]. No data exists on the ideal size of an online social network group for a behavioral intervention.

In terms of post frequency, each social media platform has norms, and it would seem imperative to match the norms of the target platform. According to one social media marketing study, the ideal frequency is 1-2 times per day on Facebook and 3 times per day on Twitter [75]. Studies using social media for behavioral interventions report a posting frequency ranging from 1-2 posts per week [17,48-50], to daily [20,68,72,76], to 2 or more posts per day [19,42,46,73,77]. The frequency of posts is likely one factor in engagement; however, given the variability in engagement across studies, the nature of posts is likely an even more important factor. The ideal post frequency may also depend on the target population for the intervention. Investigators are encouraged to solicit feedback from participants during the design phase and/or during the pilot of their intervention. Frequency of posts should be reported in manuscripts so that its association with engagement can be examined across studies.

If using commercial social networking platforms like Facebook or Twitter, intervention content can be scheduled to post in advance at a predetermined timing and frequency using social media scheduling software (eg, Hootsuite and Buffer). Scheduling software also includes features that allow you to learn the times of day users are most likely to be logged in, which can increase the likelihood of posts being viewed. A social media marketing study found that engagement rates for Facebook are 18% higher on Thursdays and Fridays, while Twitter’s highest click-through rates are on Saturday and Sunday [78]. Further, a study using Pinterest showed that articles on the topics of food and fitness are mainly posted on Sundays and Mondays [79]. These data represent average users, so ideal timing of posts may be highly dependent on the study population. Automating original posts is also helpful to keep the feed consistent and predictable, especially if new posts always appear at the same time of day. Even though posts are automated, interventionists can and should still engage with participants’ comments on those posts and attempt to draw attention to those posts via their own comments. Automation can reduce the burden of posting on the interventionist, but one downside of automation is that it may lead to interventionists forgetting to log into the community regularly. Having a log-in schedule with reminders can help to keep interventionist log-ins regular. In addition to post frequency, interventionist reaction time to participant-initiated posts matters for user engagement. According to one study, 53% who tweet to a company expect a response within the hour. If the tweet is a complaint [80], 72% expect a response within the hour. Email notifications can be set up for interventionists to make them aware of participant posts and cue them to respond.

### Group Chats

Group chats can be scheduled to conduct discussions in the same way that in-person group meetings are scheduled. Facebook has a function for conducting group chats. On Twitter, hashtags are often used to host group chats as a way to separate chat tweets from other tweets in the newsfeed and to allow people to easily follow the conversation. Chat tweets stay in the newsfeed, which allows the conversation to continue after the scheduled time of the chat. Moreover, people who missed the chat can view the chat later. Google Hangout can also be used to conduct video chats. While synchronous group chats may be more convenient than in-person meetings since they do not require transportation or childcare, they still require finding a time where everyone can attend, which may limit participation. On many platforms (eg, Twitter), group chat content can be viewed after the fact since it exists in the newsfeed. This allows participants who missed it to read the chat and comment on it later, and even allows them to reopen the discussion on a different day.

### Microcounseling

An alternative to group chats is a form of interaction we refer to as microcounseling, which involves frequent, brief, asynchronous, yet timely exchanges between an interventionist and participants [43]. In microcounseling, the interventionist logs in at least once daily to initiate and engage in discussions. Although the goal is for informational posts to elicit engagement, if they do not, the interventionist can stimulate engagement with a post that draws attention to the content by commenting on the post (eg, “I’m curious which of these strategies everyone wants to try this week?”). The interventionist can even specifically mention users in their comments to pull them into a discussion (eg, “@puppymama, you mentioned you were having trouble finding time to exercise, are any of these ideas helpful?”), similar to calling on someone in a traditional in-person group setting. Typical group dynamics emerge in online groups, such that some individuals are very talkative, while others are less so; some are advice givers, while others are advice seekers. Users of online social networks are used to fairly rapid (ie, same day) responses to their posts, thus, daily interventionist presence would seem essential to match such norms. A social media marketing study revealed that a Facebook fan page post will typically receive the majority of engagement within 3 hours [59]. Interventionists can take advantage of social media features that convey positive reinforcement (eg, “like” and “favorite” buttons) when users post and reply to increase the likelihood of such behavior occurring in the future. Another reason to address participants' comments and questions on a timely basis is the fast pace of a social media environment where posts can quickly get buried at the bottom of a newsfeed.

### Peer Influence

The engagement plan can also involve strategies to facilitate peer influence on health behavior change, potentially impacting social norms. The influence of strong ties (ie, personal connections) may be particularly important given research showing that the social norms one perceives in their friend circle may influence outcomes in behavioral interventions [81]. Other research has shown that even weak ties can be influential [82,83]. Online social networks provide a unique opportunity to engage both strong and weak ties. Peer influence can be facilitated using team-based approaches involving strong and/or weak ties [84], allowing participants to engage their friend networks (ie, strong ties) into the intervention [70], recruiting groups of friends or family members into the intervention [85], or providing corrective feedback regarding perceived social norms that may be perpetuating unhealthy behavior [71].

Peer influence can also be leveraged to spread health messages throughout large online social networks. “Viral marketing” is a marketing technique in which messages are created by an entity, but then spread within online social networks by users [86]. A recent example is the Amyotrophic Lateral Sclerosis (ALS) Association ice bucket challenge, which went viral on Facebook in the summer of 2014 and resulted in unprecedented donations for the Association [87]. This technique could be leveraged by public health interventionists to spread health messages across large networks. While it is difficult to predict which messages have the capacity to spread virally, research studying viral messages may shed light on the characteristics of messages that are shared at high rates across online social networks.

## Interventionist Training

Interventionists should ideally be experienced users of the target social media platform so that they are already acquainted with the norms of the platform. An interventionist with little or no experience with the platform would be equivalent to using an interventionist for telephone counseling who has never used or seen a phone. Training would need to be far more extensive (by an experienced user) and include review of how the device works, how people use it, and plenty of time to practice using it. Supervision should be provided throughout the intervention to flag issues. Regardless of the interventionist’s experience level, the investigator should develop a written guide for how often interventionists should log in and expectations for engagement. Although exchanges are brief on social media, conversations are continuous and dynamic 7 days a week, including holidays, weekends, and evenings. The “off hours” (ie, evenings and weekends) are also times that people changing behavior are at high risk of encountering barriers. For example, people trying to lose weight [88] or quit smoking often encounter cues in the home and social environments. Having interventionists who respond daily takes advantage of the ability to insert intervention at these times when participants need it the most. Temporary absences from the interventionist could break the flow of the conversations and result in missed opportunities to intervene. Given the need for frequent, brief attention to the group, having multiple interventionists can be helpful to cover absences/vacations as well as to model interactive engagement in a group. Participants may also prefer one interventionist’s engagement style over the other, thus, multiple interventionists reduces the possibility of disengagement from the study due to a nonpreferred interventionist.

To engage participants in discussion with interventionists and each other, informational posts alone might be insufficient, as this does not mimic typical group discussions where questions are posed and opinions are queried. Posts can be designed to engage participants into an interactive discussion by using open-ended questions, icebreakers, or conversation starters; otherwise inviting participants to respond can be used to generate discussion. In a Facebook intervention for weight loss in young adults, status updates, photos, and polls received the highest levels of engagement, with 75-97% receiving at least one interaction, while videos and links received much lower levels of engagement: 52-57% received at least one interaction [72]. Another study found that 64% of participant engagement on a study Facebook page occurred in response to the single post made by the interventionist [55]. The single post was an icebreaker, which asked participants to share experiences. In that study, the Facebook page was meant for participants to use to engage with each other, but the success of such an approach may be highly dependent on whether participants happen to feel comfortable engaging with strangers on a Facebook page. Ultimately, measuring engagement analytics throughout the course of a study will show which posts are most engaging, and this data can be used to refine the current intervention strategy in real time or in a future iteration.

## Participant Training

Even the most experienced social media users may not be accustomed to using social media to engage in a behavioral intervention. In our study of an online social network-delivered weight-loss intervention, one of the biggest barriers to engagement reported by participants was their not being sure what to post [43]. For this reason, some guidance at the outset of the intervention to inform participants on how to maximize their experience can be helpful. An orientation meeting can be held in person, by phone (eg, conference call), or online (eg, Google Hangout) to discuss the intervention and what is expected of participants. Participants can be encouraged to share their experiences, comment on posts, click the “like” button on posts they liked, and ask questions. Encouraging participants to post in the moment when they are struggling or have a question allows them to get help precisely when they need it. In addition to guidance on what to post, the orientation can give guidance on what not to post. For example, participants might be asked to refrain from posting anything for marketing purposes. On the other hand, having too many guidelines may cause participants to be inhibited from posting due to concerns about breaking rules.

When recruiting participants who are inexperienced with the social network platform, extensive training on the platform will be necessary. This would include help setting up an account, guidance regarding how to use the features and settings, familiarity with both the app and Web versions, and how to set up email notifications to cue the participant when something new has been posted or when they have been mentioned in a post. In our previous work, some participants felt that the group orientation meeting of 90 minutes was insufficient [52]. Individual meetings tailored to the participant’s level of experience might be more suitable. A run-in period during which the participant gets used to engaging regularly on the platform before the intervention begins might be useful.

## Cost and Scalability

To the extent that data prove online social network-delivered interventions efficacious, a major potential advantage could be cost-effectiveness given the elimination of clinic visits. However, implementation still requires time and effort. Potential costs to deliver the intervention include time spent setting up the community, scheduling social media posts, confirming the functionality of external links, setting up software tracking programs, interventionist/participant training, interventionist time to deliver the intervention, and other participant contact time by interventionists (eg, emails to participants who have low engagement). Studies that involve the development of a novel platform or translation of an existing behavioral intervention for social media delivery will incur additional costs related to intervention development. Investigators should take measures to track resources utilized and time spent by interventionists and participants so that cost can be accurately estimated.

The scalability of online social network-delivered interventions has not yet been explored, but would seem to have great potential given the lack of geographical barriers. Online social networks can be used to deliver interventions by health care organizations, public health organizations, and other entities that serve large numbers of people over large geographical areas. A key research question is how large can an online social network be and still effectively deliver a behavioral intervention. Another factor affecting scalability will be how much an intervention can be automated given that automation will reduce cost. Complete automation of the intervention has the highest potential for scalability, although may come at the loss of personalization. The leveraging of artificial intelligence in intervention delivery may be one way to preserve personalization. Cost-effectiveness studies are needed to truly estimate the scalability of such interventions. Researchers are encouraged to consider how their social media-delivered behavioral intervention might be scaled up for widespread dissemination and impact.

## Privacy and Human Subjects Issues

Privacy concerns can arise when using online social media platforms to deliver behavioral interventions. Privacy can be difficult to protect when using open or public settings and some people may not be comfortable engaging publicly or having others outside of the study know they are in a study [38,73]. Given that behavioral interventions are traditionally conducted in private and confidential environments, the use of private online social network communities is the best way to mimic this setting.

Investigators should make their local human studies committees and participants aware of the privacy policies of the social media platform. Because commercial platforms have access to data shared in their platform, it is not recommended that protected health information is collected over the platform, but instead via other more secure means, such as through Research Electronic Data Capture (REDCap [89]) [90]. Pretesting the group or page prior to the start of the intervention to review the privacy settings, functionality, and appearance of the content is a helpful way to identify and remedy problems before using it with study participants.

Privacy should be explained to participants at the outset of a social media-delivered intervention to ensure they understand who does and does not have access to their data. Given recent highly publicized online security breaches (eg, Target [91]), it is not only important for participants to understand the privacy settings and receive guidance on posting personal information, but it is also important for them to understand that the researchers cannot completely guarantee against a security breach.

## Engagement Data

Although engagement data—in the form of views, likes, shares, comments, favorites, replies, retweets, posts, and tweets—can be obtained by manual abstraction from the newsfeed, this can be a tedious and time-consuming task, especially for interventions with a large number of participants or long duration. A more efficient approach is to work with a programmer to extract the desired data or to use social media analytics programs to analyze the metrics. Data extraction capabilities may differ by social media platform, thus, identifying what data can be extracted and in what format in advance is recommended. Whether extracting data manually, via software, or by a programmer, proper budgeting will be important, as all require resources. Some investigators will want to analyze the content of posts made by participants, which will require capturing the text of posts so it can be analyzed. Content analyses can be conducted manually via coders or in an automated fashion using machine learning or natural language processing. Given the volume and nature of social network data, a team science approach that includes behavioral scientists, social media analysts, computer scientists, and biostatisticians is highly recommended.

## Intervention Reporting

Because the literature on online social network-delivered interventions is sparse, no reporting standards exist. Without consistent reporting about the intervention, it is difficult to compare studies and elucidate which approaches are associated with the highest engagement and success rates. Consistent reporting is also essential for replication. Table 2 outlines reporting guidelines for the intervention and for participant engagement.

**Table 2:** Reporting guidelines for social media-delivered interventions.

Intervention and participant characteristics		Reporting guidelines
Intervention general		
	Type (ie, host, user, or host and user generated)	Is the social network content intended to be host generated, user generated, or host and user generated?
	Primary modality	Is the social network the primary intervention modality or adjunctive?
	Purpose of SNS^a^	What is the purpose of the social network?
Participants		
	Experience with social media	What is the social media experience level of participants? Current users? Nonusers? Expert users?
Intervention content		
	Post frequency	How often will posts be made by the interventionists?
	Content	What is the content of the posts?
	Microcounseling	Will interventionists be providing counseling?
	Automation	Will posts be automated? If so, how many? When?
	Chats	Will moderated chats be held? If so, how often?
Participant engagement metrics		
	Likes/favorites	How many likes did each post get? On average, what percentage of posts did each participant like?
	Replies/comments	How many replies did each post get? On average, what percentage of posts did each participant reply to?
	Original posts	How many original posts did participants make? On average, how many original posts did each participant make?
Intervention fidelity		
	Page membership	What percentage of participants actually joined the group/page/community?
	Posts	What percentage of planned posts were actually posted?
	Views	How many views did each post get? On average, what percentage of posts did each participant view?
	Interventionist log-in frequency	How often did the interventionist log in?
	Interventionist likes	What percentage of participant posts/comments did the interventionist like?
	Interventionist replies/comments	What percentage of participant posts/comments did the interventionist reply or comment on?
Retention		
	Group membership termination	How many participants exited the group before the intervention ended?
	View termination	How many participants stopped viewing posts before the end of the intervention? At what point in the intervention?
	Dropout	How many participants did not attend follow-up visits?

^a^SNS: social networking site.

## Future Research

Delivering behavioral interventions via online social networks is a relatively new endeavor; thus, many questions about best practices remain unanswered. We pose several questions to be explored in future research.

1. What is the optimal size for an online social network group for a behavioral intervention? The ideal size of a social network group for each purpose (eg, microcounseling, peer support, and information delivery) is unknown. In our previous work, we found that people who tweet about their weight-loss journey reported that their organically grown social network on Twitter was, on average, 494 followers (SD 635) [40]. However, it is unclear what percentage of their network a user interacts with in organically grown social networks. Studies should explore the impact of differently sized networks on both engagement and the behavioral outcome.

2. What is the ideal structure of a group intervention? Some studies use public groups and other private groups, and each approach has its merits. Public groups allow for growth and wider dissemination of content, while private groups allow for discussions that are more intimate. The ideal structure of the group likely depends on the goal of the intervention, but this has never been explored.

3. What is meaningful engagement? Engagement comes in many forms, including hitting a “like” button, voting in a poll, or posting original content. Not all engagement may be meaningful, in other words, it may not actually result in change in knowledge, behavior, or other key outcomes. Research is needed to discern which types of engagement are associated with better outcomes.

4. How can engagement be increased in an online social network? Studies have demonstrated links between engagement and outcomes in social network-delivered interventions, but what remains unclear is how to increase meaningful engagement. Research is needed to explore the effect of group size, interventionist engagement, post type, and participant characteristics on participant engagement. The identification of modifiable factors would be particularly helpful to inform future interventions.

5. For whom are social network-delivered interventions best suited? While it may be assumed that social network-delivered interventions are best suited for regular users, the ideal way to engage nonusers is unknown. The characteristics of users most likely to benefit are unknown. People who use social media heavily to socialize may not feel comfortable, or have interest in, using social media for the purposes of learning about a health condition or changing behavior. Evaluating the target population’s interest in a social media-delivered intervention prior to attempting an intervention will likely be useful. For example, Waring and colleagues surveyed 63 overweight or obese women of childbearing age who were Twitter users to find out if they had an interest in participating in a weight-loss intervention delivered via Twitter. The majority (81%), but not all, were at least somewhat interested in such a program [92]. Further research is needed to explore which populations are most interested in this type of intervention. Replicating interventions in populations with different social media skill levels and personal characteristics, as well as using different online social network platforms, may shed light on which approaches work for whom and under what circumstances.

## Conclusions

Social media has revolutionized interpersonal communication, which presents unique opportunities for communicating with patients and delivering behavioral interventions. The design of social network-delivered interventions requires an understanding of the target platform, its users, and the norms for communication on the platform. Such an understanding will inform how the platform can be used and what role it can play in the intervention. Content from traditional interventions will require translation into a format that is consistent with how content is exchanged on the target platform. The dawn of social network-delivered interventions has also introduced a science of engagement, which requires measurement of metrics unique to each platform. Although social media presents a new means of intervening on patient behavior, many challenges and unknowns exist in the process of translating traditional intervention models for social media delivery, including the translation of intervention content, privacy, requirements and cost, and identifying the target populations most likely to be responsive. Social media research requires a team science approach that includes experts in social media analysis, behavioral science, computer science, and big data analyses. Consistent reporting of intervention details and engagement data will be crucial to advancing this science.

## Abbreviations

ALS: amyotrophic lateral sclerosis
API: application programming interface
CDC: Centers for Disease Control and Prevention
DM: direct message
N/A: not applicable
NIH: National Institutes of Health
REDCap: Research Electronic Data Capture
SNS: social networking site
